# The Future in Motion: Insights and Update from the *Journal of Functional Morphology and Kinesiology*

**DOI:** 10.3390/jfmk9010013

**Published:** 2024-01-01

**Authors:** Giuseppe Musumeci

**Affiliations:** 1 Department of Biomedical and Biotechnological Sciences, Anatomy, Histology and Movement Sciences Section, School of Medicine, University of Catania, Via S. Sofia 87, 95123 Catania, Italy; g.musumeci@unict.it; 2 Research Center on Motor Activities (CRAM), University of Catania, 95123 Catania, Italy; 3 Department of Biology, Sbarro Institute for Cancer Research and Molecular Medicine, College of Science and Technology, Temple University, Philadelphia, PA 19122, USA

## 1. Introduction

In the evolving landscape of human movement, the *Journal of Functional Movement and Kinesiology* (*JFMK*) serves as a valuable chronicle, providing a lens through which to explore our present and the perspectives for the future of human movement analysis. Our narrative begins with the pioneering experimentations in human motion, marking the genesis of our understanding. The first critical thoughts on the nature of human movement were elaborated by Aristotle in the pre-Christian era [1], but it was not until modern times that the first recorded experiment on gait analysis took place, and this historic milestone was achieved by Giovanni Borrelli in the 17th century [2]. As time progressed, the amalgamation of anatomy, physiology, and biomechanics became increasingly pronounced, culminating in the establishment of a multidisciplinary field of study, namely kinesiology. In the late 19th century, the contributions of Marey (1873) and Muybridge (1878) marked a pivotal juncture as they employed photographic techniques to quantify patterns of human movement; simultaneously, the collaboration between anatomist Wilhelm Braune and mathematician Otto Fisher resulted in measurements of body segment movements, enabling the calculation of joint forces and energy expenditures through the lens of Newtonian mechanics [3]. In today’s context, we find ourselves at the nexus of innovation, equipped with powerful tools and technologies that augment our comprehension and possibilities of analyzing complex movements. Human movement can be captured in two dimensions with photogrammetry and infrared cameras [4,5] or in three dimensions with stereophotogrammetry and inertial sensors [6]. As we delve into the contemporary landscape, it becomes evident that the symbiosis of technology and kinesiology is not confined to academic pursuits; it extends its reach into the competence of healthcare and patient well-being. The current variety of tools enhances our capabilities of recognizing not only physiological patterns but also affects the therapeutic landscape, fostering a new era of personalized interventions and tailored treatment. User-friendly systems, such as smartphones, made their debut in the field of human movement analysis with good impact, opening a new way to analyze movement with ease and with sustainable costs [7]. These tools, initially designed for coaches and trainers to evaluate athletic performance, are now gaining recognition in the broader context of movement analysis [8]. From biomechanics and wearable devices to virtual rehabilitation (the metaverse), the future holds a variety of transformative tools that stand to revolutionize the field of human movement [9]. Looking ahead, the trajectory seems even more promising thanks to emerging technologies that enable researchers and clinicians worldwide to analyze new data and investigate deeper layers of the mechanisms underlying movement. Moreover, the rising dawn of artificial intelligence is redefining the boundaries of our understanding in the field of human movement, opening new scenarios for the identification and analysis of patterns, predicting outcomes, and enhancing patient assistance. The intersection of kinesiology and technology is guiding us toward a future where the well-being of individuals is placed at the forefront. In conclusion, the goal of *JFMK* is not only to embrace the past and present but also to explore the future of human movement analysis, where the fusion of kinesiology and technological innovation promises exciting insights and advancements. Kinesiology holds the key to unlocking a comprehensive understanding of human movement, both in today’s landscape and for the future.

## 2. The Impact of the Publication of Outstanding Paper on People’s Lives

In the field of human movement studies, the impact of *JFMK* reflects the innovative visions stemming from the knowledge of anatomy and kinesiology. Distinguished by a harmonious blend of historical insights, contemporary innovations, and visionary approaches, the publication of an exceptional paper in *JFMK* showcases a deep, multidisciplinary understanding of kinesiology, movement science, and sports medicine. It integrates elements of anatomy, physiology, and biomechanics, while also embracing and pushing the boundaries of current knowledge through innovative uses of modern technologies such as machine learning models. The scope of *JFMK* is to publish papers that transcend mere academic contributions but that have a direct and tangible impact on healthcare, patient well-being, and athletes. This is reflective of the journal’s unwavering commitment to not only advancing scientific understanding but also enhancing quality of life. Papers published in *JFMK* aim to influence real-world practices and policies, offering invaluable guidance to healthcare professionals and shaping the treatment pathways for patients and training methods for athletes. They provide critical insights that lead to better health outcomes, improved movement efficiency, and an increased awareness of the integral role of movement in overall well-being.

Moreover, these papers often catalyze further research, fostering new questions and hypotheses in the field of human kinesiology. They inspire continued exploration and innovation, fostering a dynamic academic environment where knowledge is continuously evolved and refined. In this way, *JFMK* papers do not simply add to the body of knowledge; they actively transform it, changing lives by informing of new strategies for disease prevention, rehabilitation, and health promotion. A valuable paper published in *JFMK* is one that successfully bridges the gap between theoretical exploration and practical application. It makes a significant difference in people’s lives by offering new perspectives and solutions through the lens of human kinesiology, thus contributing to a broader understanding and enhanced management of human movement in various aspects of health and disease.

## 3. Looking Back on 2023

*The Journal of Functional Morphology and Kinesiology* (*JFMK*, ISSN: 2411-5142), which was first released in March 2016, developed greatly in 2023. This journal provides an advanced forum for research studies on functional morphology and kinesiology and the regulatory functions of movement. *JFMK* meets the growing demand for high-quality, peer-reviewed international journals, supplying easy access, and high publicity of open access, the digital object identifier (DOI), ORCID, and CrossRef to all researchers. We are indexed in the major research science databases, as stated on our website https://www.mdpi.com/journal/jfmk/indexing, such as Scopus (Elsevier’s abstract and citation database), the National Library of Medicine (PubMed and PMC), the DOAJ (Directory of Open Access Journals), and finally, as of this year, Web of Science (ESCI).

In 2023, *JFMK* achieved the SCImago Journal Rank with a CiteScore of 3.7 and reached Q2 quartiles in the following research fields: anatomy, histology, orthopedics, sports medicine and physical therapy, and sports therapy and rehabilitation.

*JFMK* is a member of the Committee on Publication Ethics (COPE). To verify the originality of content submitted to our journals, we still use iThenticate to check submissions against previous publications. MDPI works with Publons to provide reviewers with credit for their work and MDPI Scitations Alert to provide our authors information on new publications in their research field.

The journal publishes articles focusing on molecular, cellular, tissue, systemic, and whole-body responses to a broad definition of physical activities. Furthermore, the journal provides an advanced forum for the analysis of the structure, function, development, and evolution of the cells and tissues of the musculoskeletal system and associated clinical disorders. We are proud to inform our readers that, thanks to their continuous support, *JFMK* has continued to grow in the field of functional morphology and kinesiology research focusing on the analysis of the structure, function, development, and evolution of cells and tissues of the musculoskeletal system and the whole body. It is my pleasure to confirm the progress recorded in the last years as stated in our statistics https://www.mdpi.com/journal/jfmk/stats.

*JFMK* receives more manuscripts than it is able to publish, and the decision as to which papers are accepted or rejected is a difficult one. The decision is based on several factors, including originality, experimental design, scientific quality, data interpretation, clarity, and the quality of the written English, to maintain the high standards of our journal. As the year comes to a close, we reflect on some truly impressive statistics. Articles in the *Journal of Functional Morphology and Kinesiology* published in 2023 have been downloaded 74,964 times and viewed 263,803 times while being cited 83 times. Despite these remarkable numbers, our ongoing challenge is to further enhance the impact of the journal across various fields within the human sciences.

All articles published in *JFMK* are published in full open access format in order to provide free access to readers, and to cover the costs of peer review, copy-editing, typesetting, long-term archiving, and journal management, an article processing charge (APC) of 1600 CHF (Swiss Francs) applies to papers accepted following peer review.

In 2023, we have combined some sections of our journal to create others more attractive for the scientific community as follows:

- Mechanobiology was merged with Kinesiology and Biomechanics to create a new section entitled “Kinesiology and Biomechanics”;
- Sport Medicine and Nutrition was merged with Movement & Neurodegenerative Diseases and Motor Control and Rehabilitation to create a new section entitled “Sports Medicine and Nutrition”;
- Athletic Training and Human Performance was merged with Gait and Posture to create a new section entitled “Athletic Training and Human Performance”;
- Physical Exercise for Health Promotion was merged with Strength and Conditioning to create a new section entitled “Physical Exercise for Health Promotion”;
- Functional Anatomy was merged with Musculoskeletal Disorders to create a new section entitled “Functional Anatomy and Musculoskeletal System”.

## 4. Looking Forward to 2024

In 2024, we shall continue our efforts to improve the journal through further growth and increased visibility.

In order to achieve this target and lay a strong foundation for publications in 2024 and application for the first “Impact Factor” assessment, we have made the following plans:

- Follow-up of planned papers from editorial board members;
- Contact international conferences recommended by the editor-in-chief or by editorial board members and try to establish media partnerships with them to make *JFMK* increasingly well known among scholars;
- Communicate with editorial board members more frequently and seek their kind input and expertise for further journal development;
- Post high-quality papers on social media (e.g., LinkedIn, Twitter (now X), Mendeley, WeChat and Facebook) and increase online readership;
- Monitor the processing time of each submitted manuscript (currently 19 days);
- To be indexed by EMBASE and MEDLINE;
- To improve the Citescore in the SCImago Journal Rank in the kinesiology-related sections such as Anatomy, Histology, Orthopedics and Sports Medicine, Physical Therapy, and Sports Therapy and Rehabilitation;
- To reach the first Impact Factor released by Clarivate Analytics;
- Offer, for our authors, the best *JFMK* paper award and the *JFMK* travel grant award;
- Garner, for the sake of journal promotion, support from sponsors for our editors to participate in, and disseminate our journal to, international conferences.

From 2021, MDPI has included the accepting academic editor’s name on published articles, where they have accepted the relevant manuscript after full peer review. This supports greater transparency for our readership, demonstrates the care that our academic editors take in making decisions, and offers full acknowledgment of the effort put in when making expert judgments about a manuscript’s suitability for publication. We strongly believe that this will also support the rigorous and robust quality of our peer-review process.

We hope that you share our enthusiasm for the journal, and we look forward to working with you to make *JFMK* a leader in its field. Your contributions are vital for the success of the journal. We look forward to receiving your contributions (papers, reviews, etc.), and proposals for Special Issues are always welcome.

I wish you a healthy and prosperous new year and look forward to continuing to expand the reach and impact of the Journal with your help next year.

I would also like to take this opportunity to warmly thank, for their confidence, the following: our authors, readers, and reviewers, as well as our editorial advisors, and eminent scientists in these fields who, with their experience and important suggestions, guide us in this great enterprise; our excellent editorial board members whose depth of experience covers a very broad spectrum of different disciplines related to the morphology and kinesiology arenas; the journal’s managing editor, Mr. Jerry Wang, for his precious, professional, and daily support and the other members of the editorial office who day after day, thanks to their valuable contributions, ensure the growth of this journal; and, finally, all members of our MDPI teams in Beijing and Wuhan, China, Basel, Switzerland, Barcelona, Spain, Belgrade, Serbia, Cluj and Bucharest, Romania, and Tokyo, Japan, as well as our sponsors.
